# Respiratory Disease Occupational Biomonitoring Collaborative Project (ROBoCoP): A longitudinal pilot study and implementation research in the Parisian transport company

**DOI:** 10.1186/s12995-021-00312-4

**Published:** 2021-06-24

**Authors:** I. Guseva Canu, M. Hemmendinger, J. J. Sauvain, G. Suarez, N. B. Hopf, J. A. Pralong, T. Ben Rayana, S. Besançon, K. Sakthithasan, V. Jouannique, A. Debatisse

**Affiliations:** 1 1Department of Occupational and Environmental Health (DSTE), Centre for Primary Care and Public Health (Unisanté), University of Lausanne, Lausanne, Switzerland; 2 Division of Pulmonary Diseases, Geneva University Hospitals and Faculty of Medicine and University of Geneva, Geneva, Switzerland; 3 Autonomous Paris Transport Authority (RATP), Paris, France

**Keywords:** Subway, Indoor exposure, Particulate matter, Ultrafine particles, Metals, Biomarker, Oxidative stress, Inflammation, COPD, Cancer

## Abstract

The ROBoCoP project is launched within the EU COST Action CA16113 “CliniMARK” aiming to increase the number of clinically validated biomarkers and focused on chronic obstructive pulmonary disease (COPD) biomarker development and validation. ROBoCoP encompasses two consecutive studies consisting of a pilot study followed by a field study. The pilot study is a longitudinal exposure assessment and biomarker study aiming at: 1-understanding the suitability of the candidate biomarkers in surveying populations at risk such as workers exposed to COPD causing agents; 2-determining the best sampling plan with respect to the half-life of the candidate biomarkers; 3-implementing and validating the sampling procedures and analytical methods; 4-selecting the best suitable biomarkers to be measured in the field. Each study participant is surveyed every day during the 6–8 h work-shifts for two consecutive weeks. The field study has an implementation research designe that enabled us to demonstrate the applicability of the standardized protocol for biomarker measurements in occupational settings while also assessing the biomarkers’ validity. ROBoCoP will focus on particulate matter (PM) exposure measurements, exposure biomarkers and a series of effect biomarkers, including markers of lipoperoxidation: 8-isoprostane, malondialdehyd in exhaled breath condensate (EBC) and urine, potential markers of nitrosative stress: NO_2_^−^, NO_3_^−^ and formate anion in EBC; markers of DNA oxidation: 8-hydroxy-2’deoxyguanosine in EBC and urine, marker of genotoxicity: micronuclei in buccal cells, and oxidative potential in exhaled air (OPEA). OPEA appears particularly promising as a clinical biomarker for detecting COPD, and will be tested independently and as part of a biomarker panel. COPD diagnosis will be performed by an experienced occupational physician according to international diagnostic standards and confirmed by a pulmonologist.

This research will include approximatively 300 underground subway workers randomly selected from the personnel registry of a large Parisian transport company. Underground subways are suggested as the most PM polluted urban transport environment. We believe this occupational exposure is relevant for biomonitoring of workers and early detection of respiratory diseases.

## Background

The ROBoCoP project was developed within the EU COST Action CA16113 “CliniMARK”, which aims to increase the number of clinically validated biomarkers (https://clinimark.eu/clinimark.html) and focused on chronic obstructive pulmonary disease (COPD) biomarker development and validation [1].

COPD is a major and increasing global health problem. It is the third leading cause of death [2], the second contributing disease of disability-adjusted life-years lost [3], and the most common cause of respiratory failure. In terms of cost, the annual burden of COPD is estimated to €38.7 billion in Europe, with up to 73% of the costs related to inability to work. COPD affects 334 million people in the world, with a global prevalence of 11.7% [4]. In Europe, the prevalence of COPD ranges between 13.5 and 13.9% and is twice for men compared to women [4].

Smoking is the main but not the only risk factor for the COPD development. Occupational exposures to dusts, vapours, gases, and fumes, exposure to indoor and outdoor air pollution, maternal smoking during pregnancy or early childhood, genetic and dietary factors were recently acknowledged as COPD risk factors [5, 6]. More than 20% of COPD cases are attributable to occupational exposure [6], while for non-smokers, the estimated attributable risk for COPD associated with occupational exposure is 31% [7]. One third of COPD patients exposed to fumes and dusts have to stop working definitively due to their respiratory problems [8].

Given that occupational but also environmental exposures to particulate matter (PM) trigger both development and exacerbation of COPD [9–12] and worsens its prognosis [8], both primary and secondary prevention are paramount. However, to make the latter effective, physicians have very little useful methods available to predict and detect early disease before it clinically appears. Spirometry is the most widely used method to diagnose COPD, although it is uninformative with respect to whether the disease is a new incident or has been present for many years. Moreover, only pre-bronchodilatation spirometry is often used in routine medical examinations, rising concerns of diagnostic misclassification [4, 13]. To monitor the development of disease, sequential measures of spirometry could be helpful but are not used routinely at present [13]. Biomarkers monitoring could help in identifying individuals with healthy lung function that will develop COPD is considered promising and encouraged with respect to the secondary prevention needs. Moreover, considering the poor screening performance of spirometry, the time it takes for one test (20–90 minutes) and consequently the cost, having easy to measure, diagnostic biomarkers at disposal would be more cost-effective from a public health perspective. It will allow screening populations at risk, such as occupationally exposed workers or people living in polluted environments and targeting preventive interventions.

PM is the most important component of air pollution, and is common in occupational exposure of transport workers. Transport is an important source of PM pollution. In many cities, subway PM concentrations far exceed WHO recommended limits for 24 h average particle exposure of 50 and 25 μg/m^3^ for PM10 and PM2.5, respectively, presenting a potential risk for regular passengers and subway workers [14, 15]. Moreover, subway PM have a very particular physical-chemical composition and size distribution. In contrast with outdoor PM, subway PM is highly ferruginous, with up to 67% iron oxide (Fe2O3) in the PM2.5 mass [16] and up to 50% in the PM10 [17] and contains trace metals along with organic aerosols and minerals. PMs induce their toxicity via inflammatory and oxidative stress pathways [18, 19]. The oxidative stress mechanism plays a central role in the pathophysiology of COPD [20–22], but also of other respiratory diseases. Oxidative stress is able to induce epigenetic changes as result of direct activation of oxidative stress response genes and inflammation as result of indirect intracellular signalling pathways, through the overproduction of reactive oxigen species  (ROS) and altered gene expressions. ROS induce the cellular release of inflammatory mediators, impairing phagocytosis of apoptotic cells and weakening the ability of corticosteroids to repress proinflammatory gene expressions [23]. Inflammation, lipid peroxidation, protein and DNA oxidation can result in tissue damage, protein alteration, modified gene expression, and remodeling of extracellular matrix and mucus. The biomarkers of these effects are therefore relevant candidates for further development [19]. A large majority of biomarkers has been measured in blood [22, 24, 25] and bronchoalveolar lavage fluid [26] which require invasive sampling procedures in hospital settings. In occupational and environmental settings, safe and easily obtained biological samples are necessary [27, 28], that is why ROBoCoP is focused on non-invasive biomarkers of oxidative stress, which might be measured in exhaled air or its condensate (EBC) and urine.

The determination of the oxidative potential in exhaled air (OPEA) appears to be a novel clinical biomarker and a promising approach for detecting COPD. The OPEA analyser (EU and US patent) [29], makes the OPEA measurements available in 3 minutes and can easily be used in occupational settings. Preliminary results showed that the OPEA measured for COPD patients is significantly higher than for controls [30]. Moreover, OPEA correlates significantly with the FEV1/FVC ratio [30]. However, to be fully characterized and validated, the OPEA analyser should be assessed in different situations, and on large samples of well characterized participants. Moreover, using it within a panel of oxidative stress biomarkers may be more efficient in diagnosing COPD than a single biomarker. For that purpose, ROBoCoP focuses on a series of biomarkers including: markers of lipoperoxidation: 8-isoprostane, malondialdehyde (MDA) in EBC and urine [31–33], markers of nitrosative stress: NO_2_^−^, NO_3_^−^ [32] and formate anion [34, 35] in EBC; markers of DNA oxidation: 8-hydroxy-2’deoxyguanosine in EBC or urine and OPEA.

For implementing this trial in the field, we chose an occupational setting of the Parisian transport company (45,000 active workers), and particularly its underground subway, as subway indoor air was reported the most PM polluted urban transport environment [36–40].

## Methods/Design

### Aims and Design of the Study

The project encompasses two consecutive studies consisting of a pilot study followed by a field study.

The pilot study is a longitudinal exposure assessment and biomarker study, serving as an orientation to the field study. This pilot study aims to: (1) understand the suitability of the candidate biomarkers in surveying populations at risk such as workers exposed to COPD causing agents; (2) determine the best sampling plan with respect to the half-life of the candidate biomarkers; (3) implement and validate the sampling procedures and analytical methods; (4) select the best suitable biomarkers to be measured in the field study. The pilot study is planned to follow each study participant daily during the work-shift for two consecutive weeks. The field study has an implementation research design. Within the context of field trials, implementation research focuses on “optimizing the delivery of existing interventions that have previously been shown to be efficacious when implemented well” [41]. In our context, the field study will enable us to demonstrate the applicability of the standardized protocol for biomarker measurements in occupational settings while assessing the biomarkers validity.

### Research Setting and Participants

The research will be conducted in a Parisian urban transport company in France for the experimental part and at Unisanté in Switzerland for the analytical part. The study samples will comprise three categories of underground subway workers: locomotive operators, security guards, and station agents in charge of information, ticket sale and control. All these professionals have their workstations underground. These jobs are considered the most exposed to PM compared to other professionals working outdoor, such as bus or tram drivers, controllers and administrative staff. They are also less concerned with other chemical exposure compared to maintenance workers.

The pilot longitudinal study will use a convenience sample of nine workers; three workers per occupation. Both women and men aged 40+, and non-smokers for at least 10 years (to avoid interference of smoking with the exposure-biomarkers-health outcome relationships) will be considered eligible. The only exclusion criteria are a counter-indication of spirometry test and acute or chronic morbidities other than COPD. The participants will have their workstations on subway line 7. This line is entirely underground, deep, has no mechanical ventilation and therefore represents one of the worst case scenarios in terms of exposure and one of the best for setting a pilot study. It offers a possibility to set up rooms for participant reception, biological sampling and equipment storage. The workers recruitment will be managed by the medical coordinator of the study. The medical coordinator will organise internal company meetings with workers, their supervisors and occupational physicians. At these meetings, workers will be informed on the objectives of the pilot study, inclusion criteria and invited to contact their occupational physician to declare their wish to participate in the study and to arrange an individual interview for this purpose. The occupational physicians will check the inclusion and exclusion criteria and collect the signed written informed consent form eligible workers. The recruitment of workers will be carried out in chronological order: The eligible workers who volunteer first will be given priority.

For the field study, we will include both male and female adults regardless their smoking status. The only exclusion criteria will be a counter-indication of spirometry test (e.g., recent surgery to the head, chest, stomach, or eye, unstable angina, excessive hypertension, or a recent myocardial or stroke). We will construct a probability sample maximizing the number of workers at risk with regard to the primary health outcome (COPD). For this, an automatic stratified randomization procedure will be applied on the register of 10,778 underground workers prepared by the company’s human resources department. The strata will be defined by four variables: sex, age, smoking status (smokers, ex-smokers, non-smokers) and exposure (depending on the occupation: station agents, security guards and locomotive operators). This sample will comprise approximatively 300 workers who would provide a signed written informed consent for participation, answer the epidemiological questionnaire and participated in the medical check and biological sample collection, as described further. The sample size was calculated assuming a 13.7% prevalence of COPD [4] and the statistical distribution of the OPEA values corresponding to the preliminary study conducted in a clinical setting [30]. The sample of 300 participants will allow discriminating with 90% power COPD cases from the controls with an area under the ROC curve of 0.64 or 0.70. These values correspond to an absolute or a relative difference between OPEA and the oxidative potential of the indoor air, respectively. If including 400 subjects, these will be respectively 0.66 and 0.72. Thus, we decided to limit our study to 300 participants. Assuming a participation refusal rate of 25%, a first sample of 400 workers will be contacted by their occupational physician. Eligible and voluntary workers, estimated to 300 participants approximatively, will be enrolled, after providing a written consent.

### Exposure and Health Outcome Measures

**Fig. 1:**
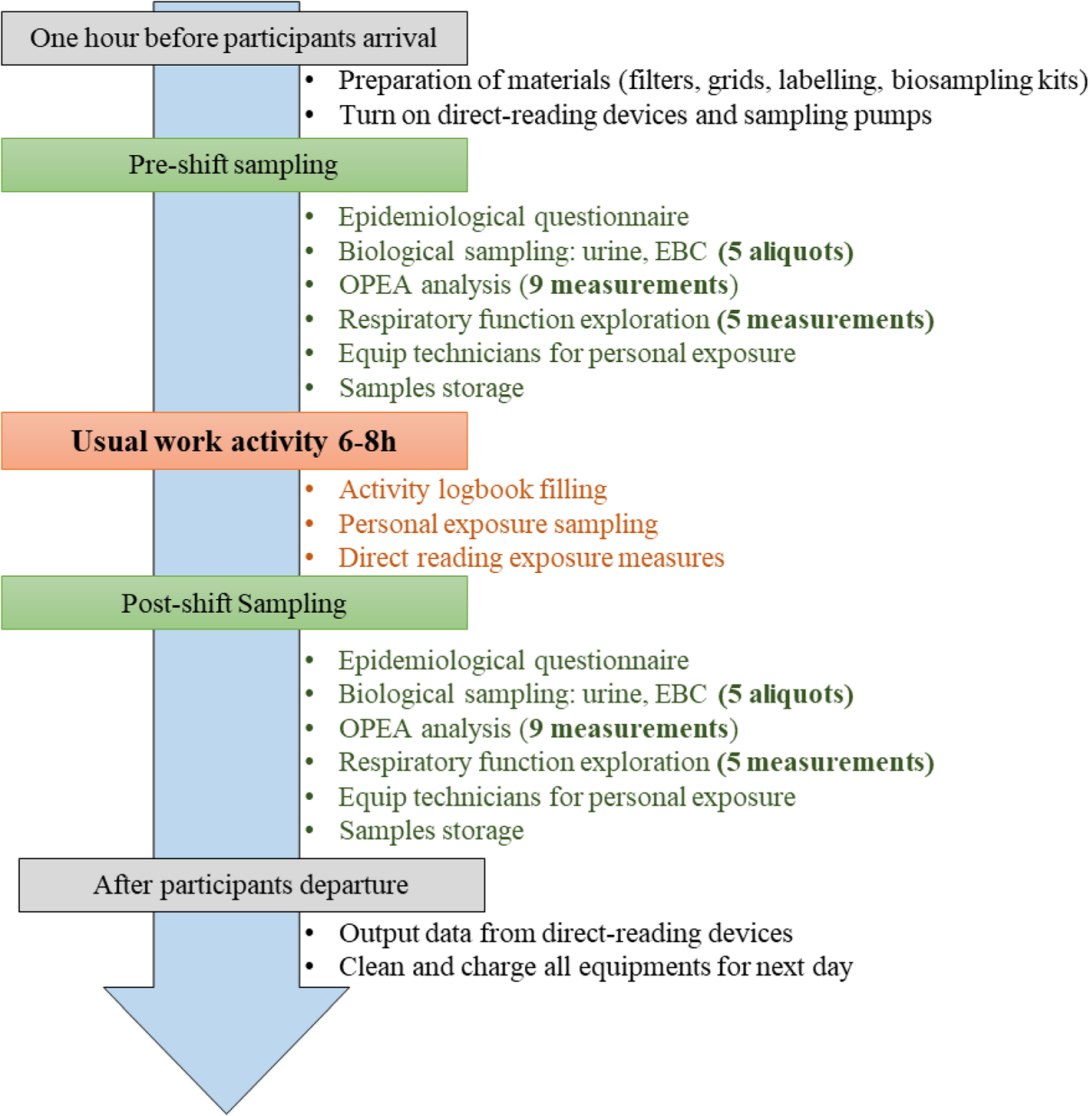
Preparatory actions and measurements carried out every day for each participant during two-week prospective follow-up in the pilot study

In the pilot study, both exposure and health outcomes will be measured. Moreover, all workers will fill in the standardized questionnaire to provide sociodemographic, occupational, life-style, and health data. Biological sampling is planned twice a day (pre-and post-shift), while exposure to PM and other pollutants possibly present in subway will be measured over the working shift. Table 1 summarizes all airborne exposure measurements, while Table 2 summarizes exposure and effect biomarkers, biological matrices and corresponding chemical analysis required. We will use Turbo-DECCS - Medivac device for EBC collection (20 minutes), Tedlar bags with Medivac vial for exhaled air collection (2 minutes), urine sampling kits for urine collection (< 5 minutes). The pilot study is scheduled for six consecutive weeks, to allow a two-week follow up for every occupation. Three workers with the same occupation will be received at the same time an hour before their work-shift. During this time, the participant will undergo a medical check including spirometry and the biological sample collection, as depicted in Fig. 1. At the end of the medical check, the workers will be provided with the air sampling pumps and direct-reading devices for monitoring airborne exposure during their working-shift. After the working-shift, participants are received again for the post-shift medical check including spirometry and biological sample collection. All spirometry tests will be performed without bronchodilation by the same trained nurse. This standardized sequence of collecting samples, taking measurements and recording data is repeated for every worker daily for two weeks. It is worth mentioning that personal air samples will be taken over the entire workday, including periods of service and breaks. A laboratory technician assigned to each participant will wear all personal sampling pumps and devices because the participants themselves are not allowed to wear any devices that can potentially interfere with their job. Moreover, each technician will fill in the workers’ activity logbook to document all its tasks, their duration, and the place of their realization during each work-shift. These logbooks will thus provide contextual information for linkage with personal exposure measurement data.

**Table 1:** Indoor air exposure measurements performed in the longitudinal pilot study in a Parisian subway

Outcome measured	Aerosol fraction	Type of measurement	Sampling method (pump and filter/head)		Sampling duration	Analytical method
Fine particle mass concentration	Inhalable (PM_10_, PM_2.5_)	Personal	3 pumps at 4 L/min	Teflon filter	8 h	Gravimetry (weighs before and after)
Metals (Fe, Cr, Cd, Al, Ba, Ni, Zn, Cu, Pb, Sb, Mn, As) mass concentration	Inhalable (PM_10_ and PM_2.5_))	Personal	2 pumps 2 L/min	Quartz filter	8 h	The metals present in particles are collected on a filter with a diameter varying from 47 to 150 mm, then dissolved in an acidic medium using a microwave mineralizer (closed system). The liquid sample is then diluted and analyzed by ICP-MS
Organic carbon/elemental carbon mass concentration	Respiratory (PM_4_)	Personal	1 pumps 2 L/min	Quartz filter - cassettes and cyclones + GALVIN (sealing filter for the cassette)	8 h	The analytical technique includes thermo-optical processes for the separate determination of EC/OC contents, based on the successive and controlled combustion of the different particles deposited on the filters, according to a given temperature program, with optical correction of scale deposits.
Monocyclic Aromatic Hydrocarbons	Gaseos	Personal	1–2 pocket pumps 0,2 L/min	Activated charcoal	8 h	(Benzene, Xylene (m, o, p), Toluene, 1,2,4 TMB)
Oxidative potential	Inhalable particles and gaseous	Personal	1 pump 2 L/min	Teflon filter (and storage cassette) + XAD2 adsorbent	8 h	1) immersion of the filter in Fox 2) chemical desorption in dichloromethane - solvent evaporation - recovery in DMSO - injection in FOX
Ultrafine particle count, size and LDSA	Respirable (PM_0.1_ (10 nm–300 nm))	Personal & stationary	5 DISCmini	impaction head	6-8 h	DISCmini, a portable personal direct-reading exposure monitor with a time resolution of 1 s.
Particle morphology and elemental composition	Respirable (PM_1_ and PM_0.1_)	Personal	1 pump 0.2 L/min	1 mini-Sampler (holding head) + grid filter (SEM)	10 min	Scanning electronic microscopy coupled with EDX (diffraction and impaction of x-rays on particles)
Fine and ultrafine particle mass concentration	PM_10_, PM_2.5_, PM_1_ (300 nm-32 μm)	Personal	1 GRIMM	Impaction head	8 h	Direct-reading optical particles counter Grimm 1.109, 31 channels, with a time resolution of 10s.
Volatile organic compounds		Stationary			8 h	Direct-reading device Gasmet
Hygrometry and temperature		Personal & stationary	2 Ecolog (ELPRO®)		8 h	Direct-reading device Ecolog (ELPRO®)

**Table 2:** Biomarker measures performed in the longitudinal pilot study in the Parisan subway

Outcome measured	Biological matrix	Analytical method
***Biomarkers of exposure***		
Transition metals (Fe, Cu, Zn, Mn, Co, Cr, Ni, Mo, Ti,V), Ba, Sb, S, Si, Al, Pb mass concentration	EBC	ICP-MS
Nanoparticles number concentration and mean hydrodynamic diameter	EBC	Nanoparticle Tracking Analyser (Malvern)
Transition metals (Fe, Cu, Zn, Mn, Co, Cr, Ni, Mo, Ti,V), Ba, Sb, Si, Al, Pb mass concentration	Urine	ICP-MS
PAH metabolites (1-hydroxypyrene)	Urine	HPLC-Fluo
***Biomarkers of effect***		
OPEA	Alveolar and bronchial part of the exhaled air	OPEA analyser + FOX colorimetric test (6 min including sampling)
Fractional Exhaled Nitric Oxide (eosinophilic inflammation)	Exhaled air	Direct-reading instrument
8-isoprostane, malondialdehyde (lipid peroxidation)	EBC	HPLC-MS/MS
8-hydroxy-2’deoxyguanosine (DNA-oxdation)	EBC	HPLC-MS/MS
NO2^−^, NO3^−^ and formate ion (nitrosant stress)	EBC	Ion-chromatography
Acetate, pyruvate, lactate, butyrate, propionate (cell metabolism)	EBC	Ion-chromatography
Malondialdehyde	Urine	HPLC-MS/MS
8-isoprostane and 8-hydroxy-2’deoxyguanosine	Urine	UPLC-MSMS
Creatinine	Urine	LC-MS/MS
Micronuclei (genotoxicity)	Buccal cells	Buccal micronuclei cytome assay

**Fig. 2:**
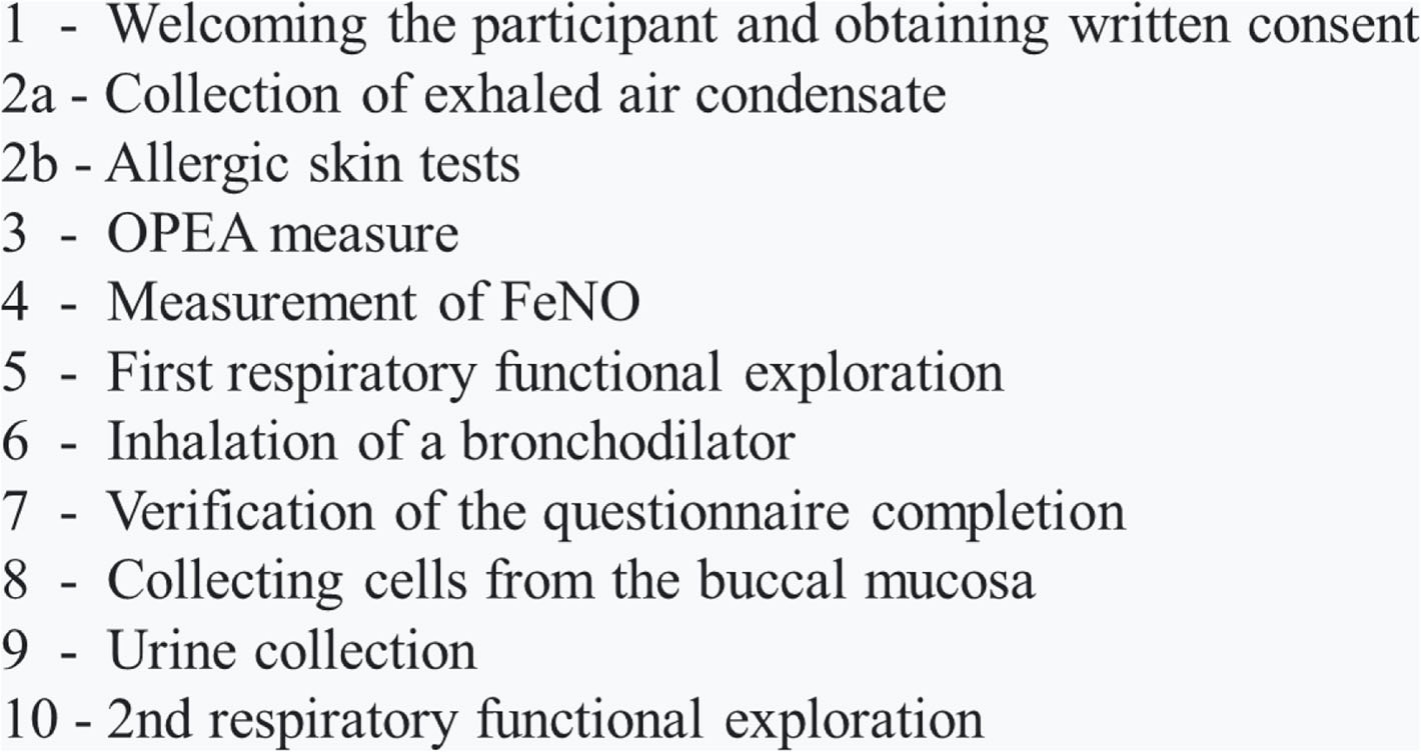
The course of the typical medical visit in the field study. *Different steps are numbered in order of completion*

In contrast to the pilot study, the field study will focus only on the biomarkers pre-selected in the pilot study as well as health outcomes, which will be measured only once per worker. All workers will be asked to fill in a standardized questionnaire, very similar to the questionnaire used in the pilot study. COPD diagnosis will be the principal clinical outcome. COPD assessment will be based on the 2017 update of the Global Initiative for Chronic Obstructive Lung Disease (GOLD) Guidelines [42]. Here, COPD is defined by incompletely reversible airways obstruction—that is, a ratio of the post-bronchodilator forced expiratory volume in 1 second to the forced vital capacity (FEV1/FVC ratio) of less than 70% in presence of the clinical symptoms such as dyspnea, chronic cough or sputum production. The GOLD guidelines recognize that the use of fixed FEV1/FVC ratio values will result in more frequent COPD diagnosis in elderly, and in less frequent diagnosis among adults younger than 45 years, especially for mild COPD. The lower limit of normal (LLN) values of FEV1/FVC, based on the normal distribution, classify the bottom 5% of healthy population as abnormal [42]. Considering the age-distribution within the cohort of the Parisian transport company workers [43], we will use the LLN values of FEV1/FVC as a second definition of COPD. For these LLN values, spirometric reference values determined from multi-ethnic reference values for spirometry for the 3–95-year age range (the global lung function 2012 equations) will be used [44]. The same well-trained occupational physician will perform spirometry to avoid inter-assessor differences. For every obstructive syndrome suspicion, a reversibility test by bronchodilation will be performed, to make a differential diagnosis between COPD and asthma. This and secondary health outcomes of interest are described in Table 2. The course of the typical medical check for the field study is depicted in Fig. 2. We will use the same methods for EBC, exhaled air and urine collection as those used in the pilot study and two single use cytobrushes (Cepilo cervical cell sampler, Deltalab S.L.U., Spain, cat. No. 440150) for harvesting buccal cells (< 5 minutes) (Table 3).

**Table 3:** Primary and secondary health outcome definition and measurment in the field study in the Parisan subway

Outcome	Measurement method	Diagnostic criteria
Chronic Obstructive Lung Disease	Spirometry performed by the trained occupational physician with the reversibility test if necessary	Post-bronchodilator forced expiratory volume in 1 s to the forced vital capacity (FEV1/FVC ratio) of less than 70% in presence of the clinical symptoms such as dyspnea, chronic cough or sputum production
Micronuclei frequency	Micronuclei counting (per 1000 cells (‰)) according to the standardized protocol (Buccal Cell Micronuclei Assay)	The individual measured values will be interpreted using a frequency diagram of micronuclei versus age in the healthy unexposed population (51). Values above reference values for a given age will be considered an early signal of the effect of genotoxic exposure
Asthma	Self-administered questionnaire	Being on treatment for asthma or at least one of the symptoms suggestive of asthma (asthma attack(s), wheezing in the chest, difficulty in breathing, attack of breathlessness) in the past 12 months
Chronic bronchitis	Self-administered questionnaire	Productive cough for at least 3 consecutive months per year and for at least 2 consecutive years or when the diagnosis has been confirmed by a physician
Emphysema	Self-administered questionnaire	Diagnosis has been confirmed by a physician
Active allergic rhinitis	Self-administered questionnaire	Symptomatic allergic rhinitis (sneezing or a runny or stuffy nose without having a cold or the flu) and under treatment during the last 12 months
Eczema	Self-administered questionnaire	Eczema diagnosis and/or treatment in the past 12 months
Atopy	Immediate reading allergic skin tests by the prick-test technique for 12 most common pneumallergens	The test results will be read by the occupational physician and considered positive if the diameter of the papule formed is greater than or equal to 3 mm and at least equal to half of the papule of the positive control (histamine)

### Statistical Analysis

Data collected within the pilot study will be analyzed using an exploratory approach. Descriptive analysis will give information on the cumulative trends of exposures to different PM fractions, their metal content and other airborne chemicals. Direct-reading fine and ultrafine particle exposure measurement data (from DISCmini and Grimm, Table 1) will be analyzed along with data from the activity logbooks, using time series analytical technics such as Bayesian spline analysis to get insights on the exposure sources and determinants.

Repeated data will be analyzed using mixed linear models and interval regression models for the censured data (below LOD/LOQ). Air and biomarker concentrations will be compared between occupations, using daily, pre- and post-shift log-transformed mean values with and without adjustment for variables collected through the standardized questionnaire (e.g., age, average home-to-work commute time, anti-oxidant diet, non-occupational exposure to PM). Correlation between external and internal exposure (based on urine and EBC biomarkers of exposure), internal exposure and early effect biomarkers, and the latter with FEV1/FVC will be tested, using post-shift to pre-shift values ratios for each biomarker. Analysis will be conducted applying different lag-times on the exposure estimates (e.g., 6 h, 14 h, and 24 h) to explore the temporal variation in biomarker levels. The most informative biomarkers (i.e., correlated both with exposure and particularly with FEV1/FVC ratio) will be selected for the field study.

**Fig. 3:**
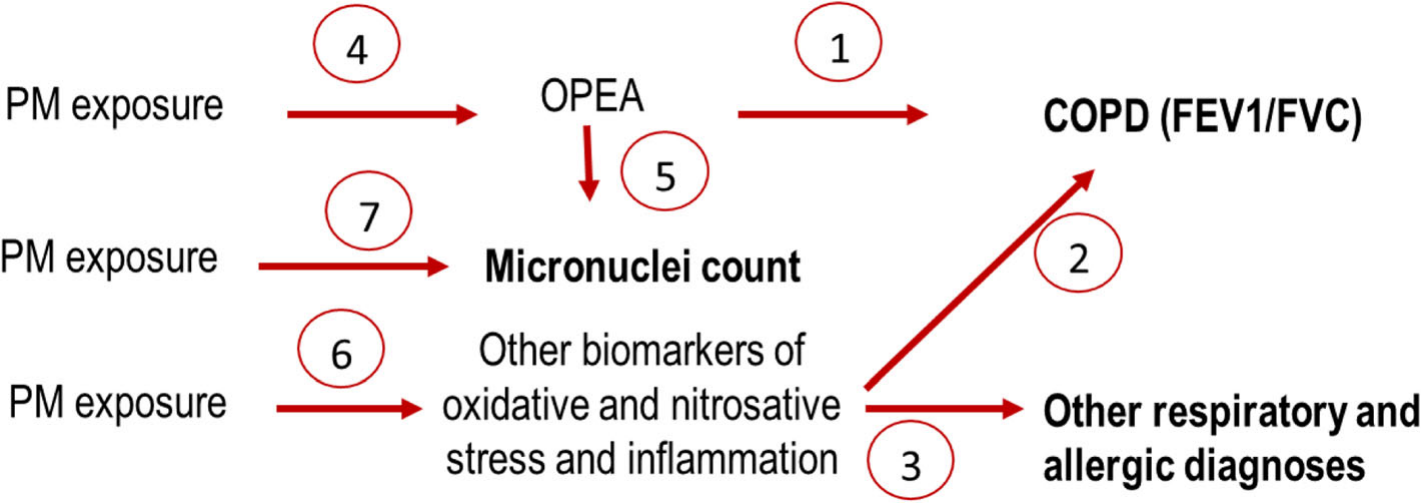
Associations to be considered during statistical analysis of the field study data, numbered by order of investigation. *The dependent variables corresponding to the studied biological or health effects are shown in bold*

In the field study, the central research hypothesis is that a panel of oxidative stress biomarkers measured in non-invasive samples, including OPEA will be more efficient for COPD diagnosis than single biomarker analysis. This hypothesis will be tested using generalized linear models. Besides, additional associations between dependent variables and explanatory variables will be examined, as shown in Fig. 3. Data management and statistical analyses will be performed using Stata, version 16, software.

### Project Temporality and Duration

The pilot study started in October 2019 and is still ongoing. Some of the laboratory analyses are very time-consuming. Most of data have already been centralized. We have started some statistical analysis.

The field study is scheduled for 2021. The campaign of individual medical checks, biological sample and data collection will last until May 2021, with a maximum duration of the medical check of 2 hours per participant. Participants will receive their individual exam results as soon as they are available and validated, by September 2021. The data will be analyzed at the end of 2021 and the first study results will be communicated in 2022. The total duration of the field study is therefore maximum of 24 months, including the experimental field phase with a duration of three months.

## Discussion

The strength of this project lies in its multidisciplinary Franco-Swiss collaboration between a university research center and a big company, involving occupational physicians, pulmonologist, toxicologists, epidemiologists, chemists, and occupational hygienists.

The second strength is the originality of the field study and its  implementation research design, owing to the preparatory longitudinal study. The extensive exposure assessment in the pilot study over six weeks is the third strength of the project. This study will help in characterizing determinants that have a short-term effect on the measured biomarkers. The diversity of exposure biomarkers and exposure metrics will give information on the interplay between external and internal exposures and the most relevant biomarkers with respect to the health effects studied. Recording exposure determinants in the activity logbook for three types of subway professionals will help with focusing the future exposure preventions so that they have the greatest potential impact on workers’ health. Finally, the physiopathological mechanisms of PM in underground indoor air and related early effects will be explored.

The field study is hypothesis-driven and will give insight on the diagnostic relevance of the biomarkers pre-selected in the pilot study, which will be tested as a panel versus OPEA alone.

The noninvasive biomarker panel is another strength of this project. In contrast to urinary biomarkers, and to a lesser extent FeNO, which are often used in occupational epidemiology, EBC biomarkers and micronuclei in buccal cells are not routinely used yet. However, these biomarkers have shown great promise in earlier work from our research group [45–49]. Thus, the biomarkers developed in this project could potentially be used in exposure monitoring and respiratory disease screening.

An important aspect of our study is that the COPD and PM exposure relationship we explore are in two different time frames. The relationships between the air exposure measurements and the biomarkers, be they measured in exhaled air, its condensate or in urine within our pilot study, reflect short-term relationships. COPD and other respiratory and allergic diseases considered in the filed study reflect the second time frame, which is chronic. Neither the respiratory symptoms nor the micronuclei frequencies are assumed to vary in the two-week pilot study. These outcomes are therefore obtained only once, in the field study. When analyzing these outcomes as a function of exposure, we have to consider long-term PM exposures (Fig. 3), although long-term PM exposure assessment is beyond ROBoCoP. The latter is necessarily less precise than the measured exposure because it has to be assessed using the jobs’ histories recorded in the standardized questionnaire. However, it represents an important research avenue, as little is known on the long-term health effects of PM in the underground environment [15, 50], given that their physical-chemical properties are very different from the PM in outdoor air pollution [14, 51]. The knowledge on ultrafine particle exposure and effects in subway workers is even more limited [15, 51]. Therefore, ROBoCoP and its ancillary studies will be informative on these knowledge gaps.

The constraints of our pilot study protocol entailed a very intensive field data collection from no more than nine participants, with one physician, one nurse, two PhD students, and a support team of five technicians present in the companies over six weeks. Given the diversity of exposure metrics and measurements and devices available it was hardly possible to include more participants. This drawback of the very complete exposure assessment in the pilot study, will be compensated with a large probabilistic sample in the field study. The latter is expected to have 90% statistical power to discriminate COPD cases from controls based on a quite small OPEA difference. On the other hand, a possible lack of power induced by the relatively small number of workers included in the pilot study will be compensated for by the exposure variance when including both highly exposed and less exposed workers, such as locomotive operators and station agents, respectively. It is noteworthy that the repeated measure design (twenty measurements per participant for urine and EBC) will contribute to increase the power of detecting short-term effects.

Bacterial contamination and endotoxin measurements were not included in the protocol due to limited resources, but also because these exposures do not raise any particular concern at the company. However, the COVID-19 pandemic has strongly affected the working conditions of subway professionals and possibly the respiratory health of those who have contracted this disease. If the pandemic continues, then this situation is likely to be an issue for a timely start and progress with the field study. To guaranty the participant and staff safety, the planning and logistics of worker medical checks should be revised. This may delay the sample and data collection process, but also decrease the participation rate. Indeed, additional time, equipment and human resources will make the field study more expensive.

In contrast, we remain confident regarding the result interpretation, even in participants who have experienced COVID-19 infection. First, the history of recent (in last 3 months) respiratory diseases will be recorded in the standardized questionnaire, and confirmed by the occupational physician during the medical visit. Second, a study conducted in parallel in Lausanne, Switzerland, aiming at comparing the background values of OPEA in a representative sample of general population (*n* = 400) according to the COVID-19 serology and lung function [52] will give insights on how to interpret values measured in the subway workers.

A careful interpretation of individual results before they are given to each participant is another important aspect of this project. For this, a tight collaboration with the company’s 34 occupational physicians is paramount. They will deliver and when necessary, explain the individual results of all measured outcomes to the participants at a medical visit scheduled after each study. To facilitate the understanding of the results, participants of the pilot study will also receive aggregated results for the study sample and possibly for their occupation. Whenever available, occupational exposure limits, indoor air quality standards, and reference values [53–58] will also be provided. Besides, the results of statistical analysis will be presented at the internal company meetings, scientific conferences and through publications in company and peer-reviewed journals. The communication of the results within the company is extremely important, as it plays a federative role between different departments and hierarchy levels and foster collaborations between occupational physicians and researchers.

## Data Availability

Data sharing is not applicable to this article as no datasets were generated or analyzed during the current study.

## Abbreviations

COPD: chronic obstructive pulmonary disease
GOLD: Global Initiative for Chronic Obstructive Lung Disease
EBC: exhaled breath condensate
EC: elemental carbon
EU COST: European Cooperation in Science and Technology
FeNO: fractional exhaled nitric oxide
FEV1: forced expiratory volume in 1 s
FVC: forced vital capacity
ICP-MS: Inductively Coupled Plasma Mass Spectrometry
LDSA: lung deposited surface area
LLN: lower limit of normal values of FEV1/FVC
LOD: limit of detection
LOQ: limit of quantification
MDA: malondialdehyde
OC: organic carbon
PM: particulate matter
ROS: reactive oxygen species
SEM: Scanning electronic microscopy
